# PROT-ON: A structure-based detection of designer PROTein interface MutatiONs

**DOI:** 10.3389/fmolb.2023.1063971

**Published:** 2023-03-01

**Authors:** Mehdi Koşaca, İrem Yılmazbilek, Ezgi Karaca

**Affiliations:** ^1^ Izmir Biomedicine and Genome Center, Dokuz Eylul Health Campus, Izmir, Türkiye; ^2^ Izmir International Biomedicine and Genome Institute, Dokuz Eylul University, Izmir, Türkiye; ^3^ Middle East Technical University, Ankara, Türkiye

**Keywords:** interface mutation, protein-protein interaction, interface design, EvoEF1, FoldX

## Abstract

The mutation-induced changes across protein-protein interfaces have often been observed to lead to severe diseases. Therefore, several computational tools have been developed to predict the impact of such mutations. Among these tools, FoldX and EvoEF1 stand out as fast and accurate alternatives. Expanding on the capabilities of these tools, we have developed the PROT-ON (PROTein-protein interface mutatiONs) framework, which aims at delivering the most critical protein interface mutations that can be used to design new protein binders. To realize this aim, PROT-ON takes the 3D coordinates of a protein dimer as an input. Then, it probes all possible interface mutations on the selected protein partner with EvoEF1 or FoldX. The calculated mutational energy landscape is statistically analyzed to find the most enriching and depleting mutations. Afterward, these extreme mutations are filtered out according to stability and optionally according to evolutionary criteria. The final remaining mutation list is presented to the user as the designer mutation set. Together with this set, PROT-ON provides several residue- and energy-based plots, portraying the synthetic energy landscape of the probed mutations. The stand-alone version of PROT-ON is deposited at https://github.com/CSB-KaracaLab/prot-on. The users can also use PROT-ON through our user-friendly web service http://proton.tools.ibg.edu.tr:8001/ (runs with EvoEF1 only). Considering its speed and the range of analysis provided, we believe that PROT-ON presents a promising means to estimate designer mutations.

## 1 Introduction

Protein-protein interactions (PPIs) are at the heart of any biological process, such as signaling, molecular transporting, and metabolic activities. Therefore, the disease-causing mutations are often found across protein-protein interfaces (Nishi et al., 2013). Mutation-induced changes across PPIs significantly alter the interface biophysics, thus the binding affinity of the interaction (David et al., 2012; David and Sternberg, 2015; Jemimah and Gromiha, 2020). As an example, Arg191Cys mutation in beta electron transfer protein impairs the protein’s interaction with acyl-CoA dehydrogenases (ACDHs) due to a decrease in the enzymatic activity, induced by the Arg191Cys mutation. This decrease leads to the glutaric aciduria IIB metabolic disease. (Yates and Sternberg, 2013; Henriques et al., 2021).

To gauge the binding affinity changes induced by interfacial mutations, several computational tools have been developed (Navío et al., 2019). A recent benchmarking effort has shown that among such tools FoldX (Schymkowitz et al., 2005) and EvoEF1 (Pearce et al., 2019) can predict the structural impact of a mutation with top ranking accuracies in the fastest manner (Amengual-Rigo et al., 2021). For this, FoldX and EvoEF1 structurally model the mutation under the effect of a force field, followed by the scoring of the new interface with a linear empirical energy formula (composed of van der Waals, electrostatic, hydrogen bonding, and desolvation energies, as well as entropic penalties). Expanding on the capabilities of FoldX and EvoEF1, we developed PROT-ON (PROTein-protein interface mutatiONs), which aims to deliver the most critical PPI mutations, i.e., the designer mutations, that can be used to propose new binders. For this, PROT-ON probes all possible interface mutations with either FoldX or EvoEF1 on a selected protein partner. It then statistically analyzes the scanned energy landscape and filters out the most significant affinity altering mutations according to stability and optionally evolutionary criteria. Together with this list, PROT-ON outputs several residue- and energy-based plots, portraying the synthetic energy landscape of the probed mutations. The stand-alone version of PROT-ON is deposited at https://github.com/CSB-KaracaLab/prot-on. Our tool can also be accessed *via* our user-friendly web interface http://proton.tools.ibg.edu.tr:8001/ where only EvoEF1-based calculations are performed. The web-based calculations take less than 10 min.

In this manuscript, we firstly explain the architecture of PROT-ON. We then demonstrate its use on MDM2-p53, ACE2-RBD, and MCL1-NOXA protein-protein complexes. With these proof-of-concept PROT-ON cases, we aim to guide our users in finding the most critical PPI positions in a speedy manner, which can translate into possible designer mutations.

## 2 Materials and methods

### 2.1 The PROT-ON pipeline

In the PROT-ON pipeline, the interfacial amino acids of one protein partner are identified by using the classical Euclidian distance formula (Eq. 1). By using this distance calculation, we define the interchain residues that contain at least one atom within a certain cut-off as interfacial amino acids. By default, the distance cut-off is set to 5 Å. Then, by using the relevant FoldX and EvoEF1 functions, the interfacial amino acids of the selected partner are mutated into 19 other amino acid possibilities, followed by calculating the energy score of each mutation. So, if there are 15 interface amino acids defined on one monomer, PROT-ON will calculate 15 × 19 energy scores. Then, all these predicted energy scores will be analyzed with non-parametric box-and-whisker statistics. In box-and-whisker statistics, 50% of the data around a median is defined as an interquartile range (IQR) and represented as a box. Whiskers are extended from the opposite sides of the box by multiple IQRs to cover the data spread. Any data falling outside this whisker expansion is classified as an *outlier* (Supplementary Figure S1). The negative *outliers* are defined as mutations that significantly improve the binding (enhancing mutations, ∆∆G << 0), while the positive *outliers* are the ones that significantly worsen the binding (depleting mutations, ∆∆G >> 0). We return these mutations as the initial designer mutation set. By default, the IQR threshold is set to 1.5.

$$\left|AB\right|=\sqrt{{\left(x_{A}-x_{B}\right)}^{2}+{\left(y_{A}-y_{B}\right)}^{2}+{\left(z_{A}-z_{B}\right)}^{2}}$$

Equations 2, 3. The Euclidian distance formula used to calculate the interatomic distances between two protein partners, i.e., A and B.

The initial designer mutation list is then filtered according to the stability score of each mutation. Here, if a mutation has a FoldX or EvoEF1 stability score <0, then the mutation is kept. The stable mutations can optionally be filtered out further according to a user supplied PSSM (position specific scoring matrix) file. PSSM measures the probability of observing a particular mutation at a given position. PROT-ON keeps a depleting mutation, if its PSSM score is =< 0. Complementarily, it keeps an enriching mutation prediction if its PSSM score is >0. We use these cut-offs as we observed that the most enriching and depleting mutations span such PSSM values. For more, please see Supplementary Figure S2. The mutation set remaining after the consecutive filtering steps are returned to the user as the final designer mutation set.

### 2.2 FoldX and EvoEF1 functions used in PROT-ON

To calculate the impact of PPI mutations, we use FoldX 4.0 and EvoEF1 January-2021 versions. We stick to these versions, as they are the ones that were benchmarked on the SKEMPI 2.0 data set (Jankauskaitė et al., 2019; Amengual-Rigo et al., 2021). For both tools, the input complex is supplied in the PDB format, where the first step is an energy minimization aiming at removing the interatomic clashes (commands: **RepairStructure** for EvoEF1; **RepairPDB** for FoldX). The interfacial mutations are then imposed on the energy-minimized structure on one partner (commands: **BuildMutant** for EvoEF1; **BuildModel** for FoldX). Finally, binding free energy and stability scores are calculated with the empirical energy formulas of each algorithm (commands: **ComputeBinding** and **ComputeStability** for EvoEF1; **AnalyseComplex**, **Stability** for FoldX).

FoldX estimates the free energy of binding (ΔG_FoldX_) by using van der Waals forces (ΔG_vdw_), electrostatic interactions (ΔG_el_), hydrogen bonding (ΔG_hbond_, ΔG_wb_), and solvation energies of polar (ΔG_solvP_), and hydrophobic groups (ΔG_solvH_). In addition to these, it also considers the entropy by statistically analyzing the phi-psi distribution for each amino acid (ΔS_mc,_ ΔS_sc_). The empirical formula of FoldX is given in Eq. 2 (Schymkowitz et al., 2005).

$$\Delta G_{FoldX}=a\cdot \Delta G_{vdw}+b\cdot \Delta G_{solvH}+c\cdot \Delta G_{solvP}+d\cdot \Delta G_{wb}+e\cdot \Delta G_{hbond}+f\cdot \Delta G_{el}+g\cdot \Delta G_{kon}+h\cdot T\Delta S_{mc}+k\cdot T\Delta_{Ssc}+l\cdot \Delta G_{clash}$$

Equation 2. The empirical energy formula of FoldX to calculate the binding free energy change.

EvoEF1 uses similar terms, i.e., van der Waals forces (E_vdw_), electrostatic interactions (E_elec_), hydrogen bonding contribution (E_HB_), desolvation energies of polar and hydrophobic groups (E_solv_), and reference energies of 20 amino acids to describe atomic interactions (E_ref_) (Eq. 3) (Pearce et al., 2019).

$$E_{EvoEF1}=\sum {\left[E\right.}_{vdw}{\left(i,j\right)+E}_{elec}{\left(i,j\right)+E}_{HB}{\left(i,j\right)+E}_{solv}{\left(i,j\right)]-E}_{ref}$$

Equation 3. The empirical energy formula of EvoEF1 to calculate the binding affinity of a PPI.

For both tools, the impact of the mutation is calculated by subtracting the wild type energy value from the mutant one.

### 2.3 The packages used in the stand-alone and the web service versions of PROT-ON

The standalone PROT-ON uses NumPy (https://numpy.org/) (Harris et al., 2020) to create lists and to perform scientific calculations. Pandas (https://pandas.pydata.org/) (McKinney, 2011) is used to read data frames and generate output files. Plotly (https://plotly.com/) and Kaleido (https://pypi.org/project/kaleido/) are used to create heatmap and box-and-whisker plots, as well as to generate static images of creating plots. The latest version of PROT-ON can be cloned from https://github.com/CSB-KaracaLab/prot-on for Linux and MacOS systems.

The back end of the PROT-ON web server is developed by Flask (https://flask.palletsprojects.com/en/2.1.x/) (Grinberg, 2018) which is a Python microframework. On the other hand, HTML, CSS, JavaScript languages, and Bootstrap (https://getbootstrap.com/) web application development toolkit are used to develop the front end of the web server (navigator menu, grid system, table, form, etc.). Fundamental input types like text, slider, email, etc. are written in HTML. CSS, JavaScript, and Bootstrap are used to design input types and create a grid system (container, row, columns). Celery Python package (https://docs.celeryq.dev/), RabbitMQ message broker (https://www.rabbitmq.com/), and SQLAlchemy toolkit (https://www.sqlalchemy.org/) (Brown and Wilson, 2012) are used to process submitting jobs in the background. Celery is a distributed task queue that allows the creation of different tasks running in the background asynchronously. Celery is used to prevent data accumulation in the workstation by running the scheduled task and to process submitted jobs by a worker task. Celery also communicates with a message broker, RabbitMQ (to receive and send job messages and a database) and a database, SQLAlchemy (to store results). The web server is deployed by Nginx (https://www.nginx.com/) (Reese, 2008), Gunicorn (https://gunicorn.org/), and the supervisor process control system (http://supervisord.org/). Nginx is open-source web-service software that serves as a reverse proxy, and load balancing for the HTTP web server. Gunicorn is a WSGI HTTP server written with Python. Flask communicates with Nginx by converting HTTP requests to Python. Flask requires a Supervisor control process system to start automatically if anything goes wrong. Demonstration of processing runs in the background and deployment of the web server is given in Supplementary Figure S3.

PROT-ON service is accessible over http://proton.tools.ibg.edu.tr:8001. PROT-ON is hosted on an HP Z2 Tower G4 workstation [64-bit 3.40 GHz Intel (R) Xeon (R) E-2124 G CPU with four cores and 32 GB RAM] connected to https://ibg.edu.tr. The system uses Ubuntu OS 22.04 LTS version.

## 3 Results

PROT-ON aims to analyze the mutational landscape of a protein complex to provide with potential (binding enriching or depleting) designer mutations. For this, PROT-ON takes a protein dimer in the format of a PDB file as an input, together with the monomer (chain) of interest. After this, all interfacial amino acids of the selected partner are mutated into other 19 amino acid alternatives. This procedure is followed upon collecting the energy scores of the scanned mutational landscape. The mutation energy scores are filtered according to statistics-, stability- and optionally evolutionary-based criteria. These filtering steps are designed to ensure proposing biologically feasible mutations, so that they can be utilized in constructing new protein binders. Below, we explain the framework of PROT-ON, as well as our server architecture. We also demonstrate the use of PROT-ON on three different protein-protein complexes.

### 3.1 The PROT-ON framework

**FIGURE 1:**
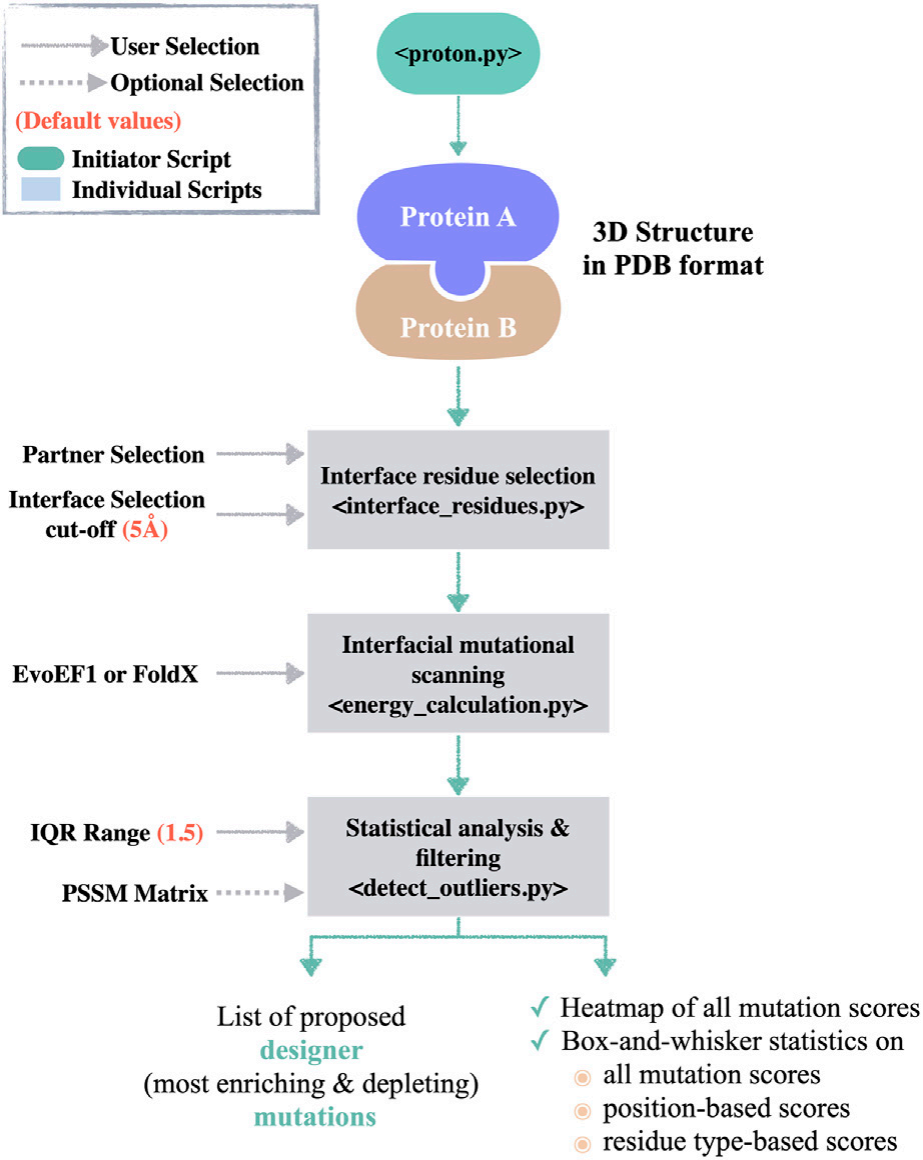
The PROT-ON pipeline. The **proton.py** calls the individual scripts, i.e., **interface_residues.py**, **energy_calculation_EvoEF1.py/energy_calculation_FoldX.py**, and **detect_outliers.py**. The default values for the user-defined parameters are denoted in salmon.

The functions of PROT-ON are called by the initiator **proton.py** script (Figure 1). When the user supplies the protein dimer structure in the PDB format, together with the chain of interest, **interface_residues.py** script is called. The **interface_residues.py** calculates the interfacial amino acids within a user provided cut-off (default is 5 Å, see **Methods 2.1**). After the selection of mutation calculation engine (either FoldX or EvoEF1), **energy_calculation_[selected_method].py** is called to scan all interfacial mutations on the selected partner. The same script also calculates the estimated binding energy changes between the mutant and wild type states (for the functions used here, please see **Methods 2.2**). This step is followed by running the **detect_outliers.py** script. The **detect_outliers.py** applies a box-and-whisker statistics on the scanned energy landscape. In box-and-whisker statistics, 50% of the data around a median is defined as an interquartile range (IQR) and represented as a box. Whiskers are extended from the opposite sides of the box by multiple IQRs to cover the data spread. Any data falling outside this whisker expansion are classified as negative and positive *outliers* (Supplementary Figure S1). Here, our assumption is that the negative *outlier mutations* will significantly improve the binding (enhancing mutations, ∆∆G << 0), while the positive *outlier mutations* will significantly worsen the binding (depleting mutations, ∆∆G >> 0). Expanding on this assumption, **detect_outliers.py** script isolates the *outlier* mutations. If needed, the user can play with the IQR range used to define the extent of whisker, thus the extent of *outliers* (1.5 by default). To ensure the selection of biophysically sound mutations, the same script filters out the isolated *outliers* according to their stability scores. Here, we provide the option to supply an external PSSM score file in case the user wants to incorporate evolutionary information into the process (see **Methods 2.1**). The **detect_outliers.py** script further generates a heatmap, as well as residue-based energy analysis to portray the energy landscape scanned by all mutations. The PROT-ON framework is presented in Figure 1. The input/output files generated by the tool are described in https://github.com/CSB-KaracaLab/prot-on.

### 3.2 The PROT-ON web service

**FIGURE 2:**
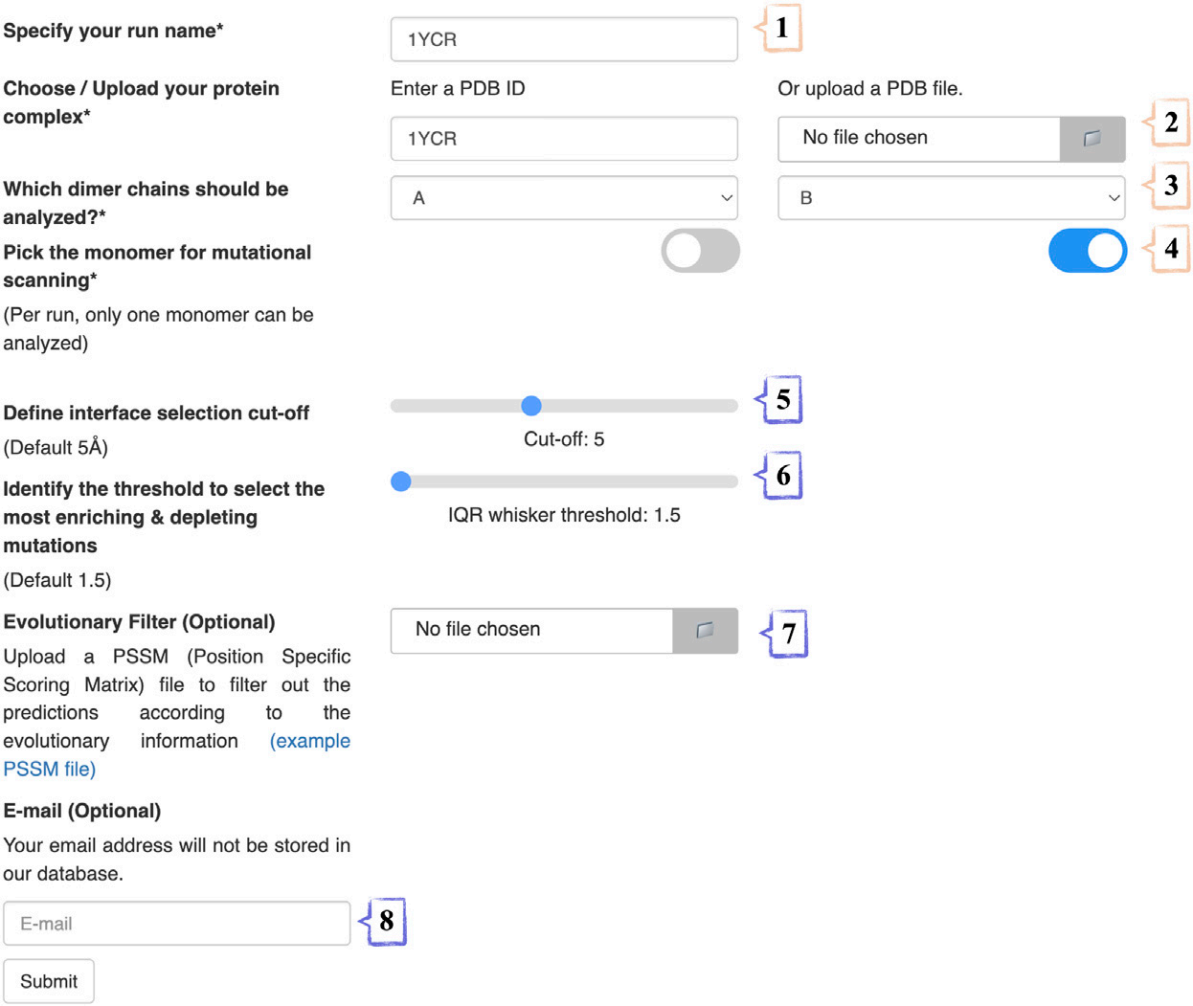
The stepwise annotation of PROT-ON run submission page with 1YCR (MDM2-p53 case) example. Wheat and slate colors represent mandatory and optional fields, respectively.

We present an easy access point to our PROT-ON framework *via* http://proton.tools.ibg.edu.tr:8001. Our web service runs only with EvoEF1, as FoldX requires a personal academic license. To submit a run, four mandatory and four optional steps should be followed (Figure 2). In the 1st step, the run name is specified. In the 2nd step, either the PDB ID of the case is inputted or the PDB coordinates of the complex is uploaded. In the 3rd step, the chain IDs of the protein dimer of interest are selected (from a drop-down menu). In the 4th step, the partner to be analyzed is indicated by switching the relevant toggle. In the optional 5th step, the cut-off distance for defining the interfacial amino acids is set (choices range from 3.0 Å to 8.0 Å, changing with an increment of 0.1 Å). In the optional 6^th^ step, the user can play with the IQR threshold to define the outliers (choices range from 1.5 to 2.5, changing with an increment of 0.5). In the optional 7^th^ step, a PSSM file in the csv format is supplied. In the 8^th^ and optional step, the e-mail address where the result link will be delivered is entered.

On the result page, a summary of the run, a downloadable run folder (stored for 1 week), three interactive energy plots, and the final designer mutation list is presented (Supplementary Figure S4). The summary section includes the number of interfacial amino acids selected within the defined cut-off distance, the total number of mutations imposed, and the number of final enriching and depleting mutations given the input IQR range. This section is followed by an interactive box-and-whisker plot of the sampled energies and their heatmap, as well as the energy distribution of each interfacial residue. The latter plot can be used to dissect the residues critical for binding. The relevant values on the energy plots can be visualized by hovering the mouse over them. The final enriching and depleting designer mutation list is presented as an interactive table where the user can focus on rows, select, and hide columns.

Below, we demonstrate the use of our web interface on two case examples, i.e., MDM2-p53, ACE2-Spike, and the stand-alone version with FoldX on the MCL1-NOXA complex.

### 3.3 Proof-of-concept server case 1: MDM2-p53 complex

**FIGURE 3:**
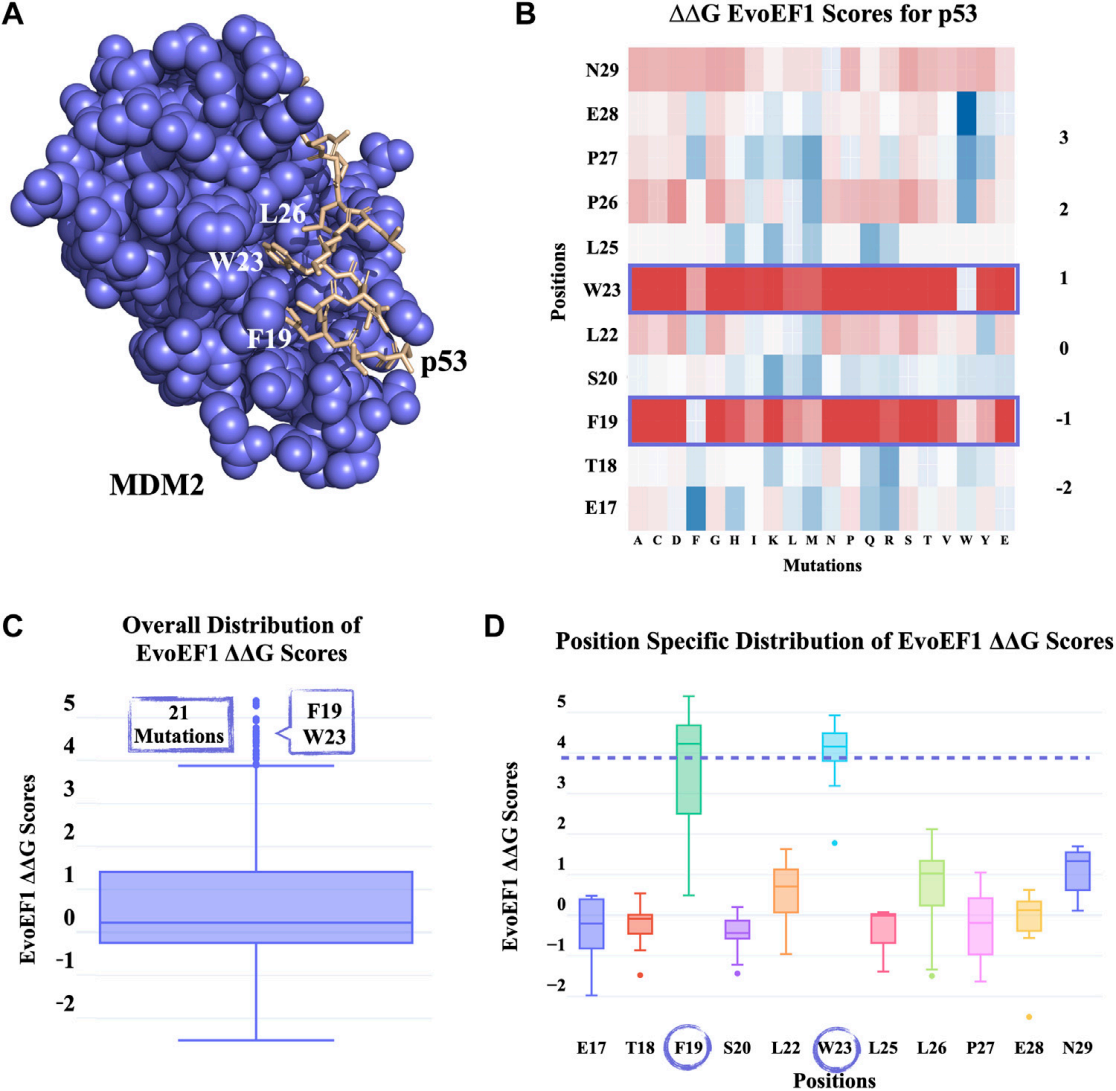
PROT-ON results on p53 from the MDM2-p53 complex with default run parameters. **(A)** Slate and wheat colors show MDM2 and p53 proteins, respectively. The critical p53 residues for the MDM2-p53 interaction are depicted in sticks. **(B)** EvoEF1 mutational energy heatmap. The detected critical positions are marked with a rectangle. **(C)** The box-and-whisker statistical analysis of the EvoEF1 mutation scores. There are 21 positive *outliers* detected by EvoEF1. **(D)** Position-specific distribution of energy scores for p53 calculated by EvoEF1. The purple dash line represents the upper whisker end value, as given in Figure 3C. The encircled residues indicate the position of *outlier* mutations.

p53 is a tumor suppressor, which decides for the apoptotic state of the cell in case of cellular damage (Moll and Petrenko, 2003). MDM2 is a regulator of p53, specifically controlling the overaccumulation of p53. At the extreme end, in cancer cells, MDM2 is often found to be overexpressed, blocking p53 activation (Nag et al., 2013). Therefore, it has been of utmost importance to cancer research to understand the key interactions between MDM2 and p53. In 1996, the MDM-p53 complex structure was solved with X-ray diffraction at 2.60 Å resolution (PDB ID: 1YCR) (Kussie et al., 1996). This structure showed that p53’s key F19, W23, L26 residues penetrate the hydrophobic cleft of MDM2 (Figure 3A). In our first proof-of-concept case, we run PROT-ON web service on p53 to probe whether we can back-calculate these crucial positions.

The PROT-ON run with default parameters leads to selection of 11 interfacial p53 residues, which results into the calculation of 209 interfacial mutations. As shown on the all energy heatmaps, EvoEF1 spots two from the three critical amino acids (Figure 3B), which is also reflected on to the box-whisker statistics and residue-based energy analysis (Figures 3C, D; Supplementary Table S1). F19D/E and W23C/K/R/T/V are proposed to be the most depleting designer mutations after imposing the stability filter of EvoEF1. Notice that, F19 and W23 are reported to reduce transactivation when mutated into charged or polar amino acids (Kussie et al., 1996). Even though PROT-ON did not propose any enriching mutations, we suggest that the lowest energy scores of EvoEF1 (E28W) still hold the potential to increase the binding affinity. This might happen by adding more hydrophobicity to the interface towards the two ends of the p53-peptide. This is also endorsed by the fact that the most enriching mutations are converted to an amino acid that has an aromatic side chain (Figure 3D). These run results can be reached at http://proton.tools.ibg.edu.tr:8001/result/MDM2_p53_EvoEF1.

### 3.4 Proof-of-concept server case 2: ACE2-spike complex

**FIGURE 4:**
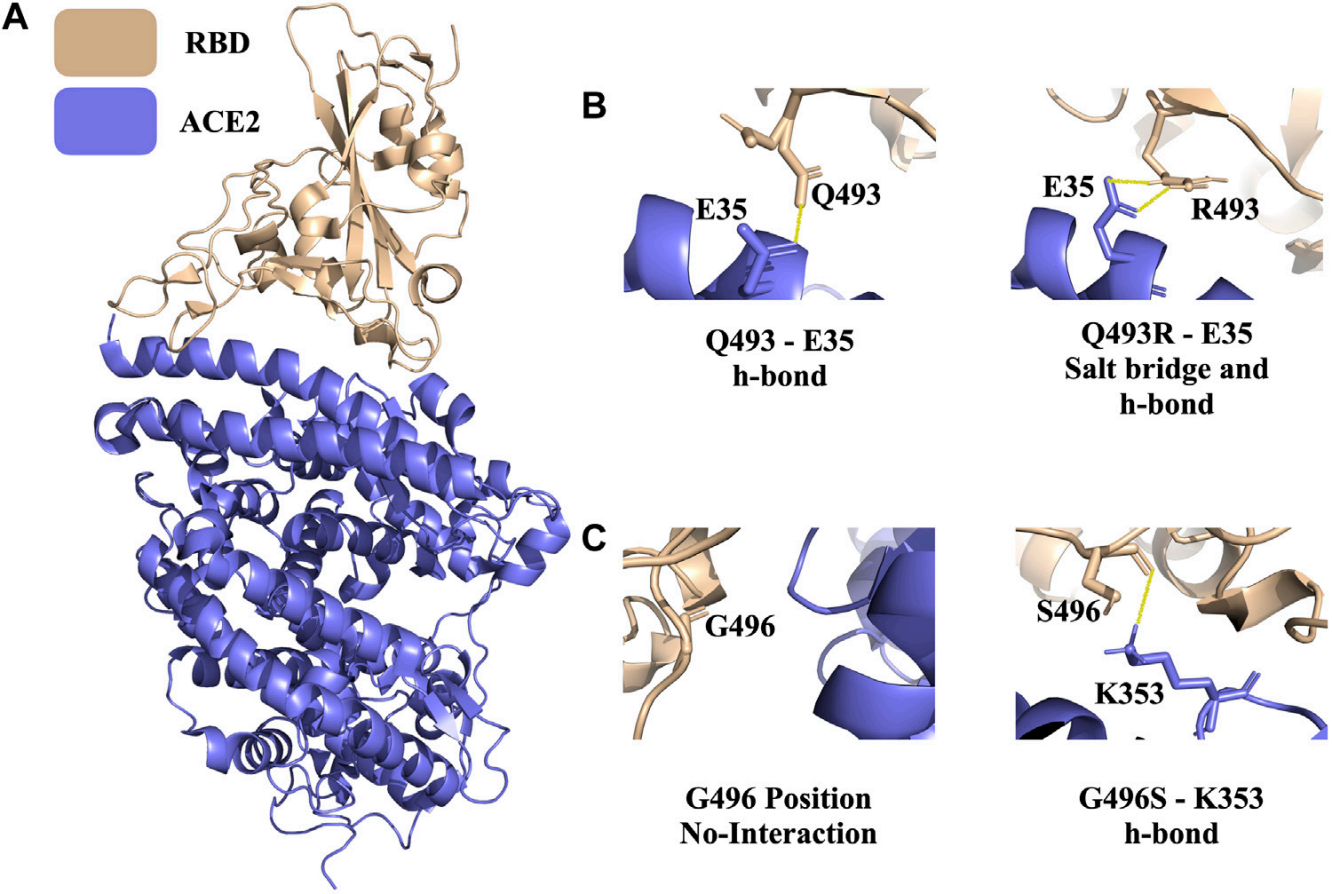
PROT-ON results on RBD from the ACE2-RBD complex with 7 Å interfacial cut-off. **(A)** The structure of the ACE2-RBD complex (PDB ID: 6M0J). Wheat and slate denote RBD and ACE2, respectively **(B)** Polar interactions of wild type Q493 and mutant Q493R are shown (solid yellow line). **(C)** The depiction of the wild type G496 and mutant G496S. The solid yellow line represents the newly formed h-bond.

Since late 2019, SARS-CoV-2, a novel SARS virus, has caused a global outbreak, leading to the deadliest pandemic of the 21st century. Since the early days of the outbreak, we know that the infection cycle starts by having the receptor binding domain (RBD) of the virus’ spike protein interacting with the host Angiotensin Converting 2 (ACE2) enzyme (Jubb et al., 2017). The ACE2-RBD crystal structure is resolved in 2020 with X-ray diffraction (2.45 Å resolution, PDB ID 6M0J) (Figure 4A) (Lan et al., 2020). Recently, Tian *et al.* performed experimental studies to investigate the role of dangerous RBD mutations, like the famous N501Y RBD mutation in Alpha, Beta, and Gamma variants. This mutation led to new π–π and π–cation interactions, improving the binding affinity between ACE2 and RBD (Tian et al., 2021). In addition, K417N, G446S, Q493R, G496S, and Q498R are some of the commonly observed interfacial RBD mutations that are seen in the circulating Spike variants (Hodcroft, 2021).

For the ACE2-RBD case, we run PROT-ON with EvoEF1 with a 7 Å interfacial cut-off (to scan a wider range on the interface) by keeping the default IQR threshold. As a result, 39 RBD interfacial amino acids are detected on RBD and a total of 741 interfacial mutations are scanned. In this case, the box-and-whisker statistics defines the previously reported Q493R, G496S, and N501Y variants as binding enhancing mutations (Supplementary Figure S5). After the stability filtering, 36 mutations are selected as the final enriching designer mutations (Supplementary Table S2), with Q493R and G496S being in this final list. Q493R leads to the formation of a new salt bridge with E35 (Figure 4B). There is also a new hydrogen bond forming upon G446S mutation (Figure 4C). PROT-ON also suggests additional mutations on the same critical residues, as observed in the common variants, i.e., K417R, G446R, Q498E/F/I/K/L/M/N/S/T/Y, and N501L (Supplementary Table S2). As a result of the structural analysis of K417R, we observe an additional salt bridge when compared to existing K417N variant. So, the final PROT-ON designer mutation set should be considered as “possibly dangerous” due to their binding enhancing effect. Our results for this case can be accessed through http://proton.tools.ibg.edu.tr:8001/result/ACE2_RBD_EvoEF1 (Supplementary Figure S5).

### 3.5 Proof-of-concept stand-alone case: MCL1-NOXA complex

**FIGURE 5:**
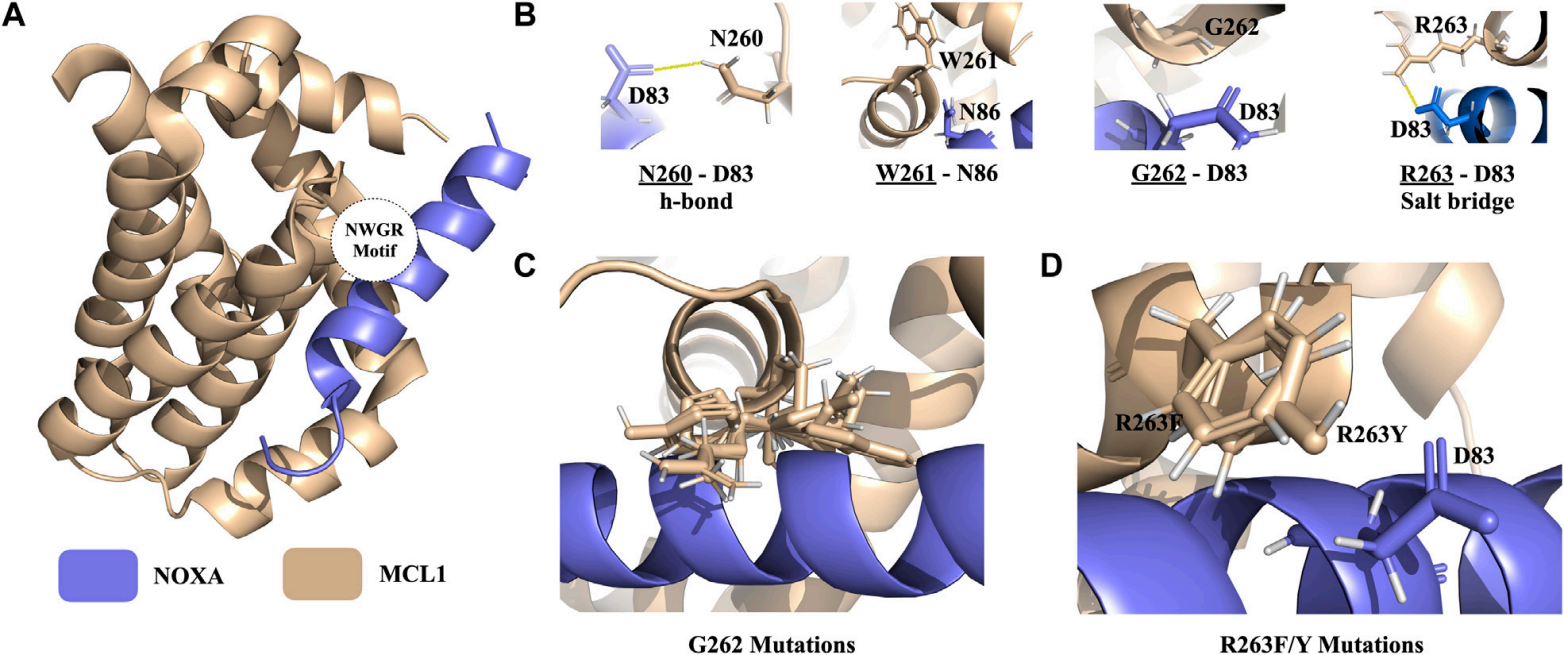
PROT-ON results on MCL1 from the MCL1-NOXA complex with 2.5 IQR threshold and PSSM input. **(A)** The structure of the MCL1-NOXA complex (PDB ID: 2NLA). The wheat and slate colors denote the MCL1 and NOXA, respectively. The positioning of the NWGR motif is encircled. **(B)** NWGR motif of MCL1 with close by amino acids shown with sticks on NOXA. The polar interactions are shown in solid yellow lines. **(C)** Orientations of the depleting mutations generated for G262. **(D)** Orientations of the depleting mutations generated for R263.

Protein-protein interaction of the B-cell lymphoma-2 (BCL) family is responsible for the regulation of apoptosis by interacting with their coregulators. This process controls mitochondrial outer membrane permeabilization and causes the release of intermembrane space proteins, followed by the activation of caspases (Xie et al., 2021). MCL1 belongs to the anti-apoptotic class of the BCL family that can inhibit apoptosis and NOXA is its coregulator (Youle and Strasser, 2008). The crystal structure of the MCL1:mNoxaB BH3 complex was identified in 2.80 Å resolution and deposited in the PDB with 2NLA PDB ID (Figure 5A) (Czabotar et al., 2007).

Ivanov et al. (2016) determined a conserved “NWGR” (N260, W261, G262, R263) motif on MCL1 (Figures 5A, B). Xie *et al.* further explored that mutations on N260 and G262 from the NWGR motif results in the disruption MCL1 and NOXA complex formation. To explore whether PROT-ON can get back the same depleting effects on MCL1, we run the stand-alone version of PROT-ON with FoldX, with the default interface cut-off, and with 2.5 IQR threshold. In this case, we also inputted a PSSM file for MCL1. As an outcome, we obtained 26 interfacial amino acids with 494 interfacial mutations. As shown on the heatmap and box-and-whisker analysis, N260, G262, and R263 come out as the most detrimental positions irrespective of the mutation type, so agreeing with the suggested importance of the NWGR motif (Supplementary Figure S6B). After stability and PSSM filtering, we are left with a final list of 28 designer-depleting mutations (Supplementary Table S3), where all G262 mutations except for C and T and R263/F/Y mutations are proposed as designer-depleting mutations. From the inspection of the G262 mutation models, it is evident that there is no space left for a bulky residue change (Figure 5C). R263F/Y causes the removal of an important salt bridge and thus has an abolishing effect on the binding (Figures 5B–D). Run results for this case can be found at http://proton.tools.ibg.edu.tr:8001/result/MCL1_NOXA_FoldX (Supplementary Figure S6).

## 4 Conclusion

Here, we present a new tool, PROT-ON, which aims to find the critical mutations to redesign known protein-protein interactions. The proposed PROT-ON mutations can be especially utilized to speed up the targeted mutation search in protein-based therapeutics. To assist such a search, PROT-ON’s server not only suggest a mutation list, but it also displays several interactive energy plots to portray the scanned mutational landscape. We showcase the capabilities of our tool on three important cases with diverse functions. These proof-of-concept cases validate known critical positions/mutations of the studied complexes. They also offer new mutations to enhance or abolish the known interactions. Our hope is that these and other cases probed by the users will set a milestone in guiding novel experimental research.

## Data Availability

The datasets presented in this study are provided as online links in the Results section. The stand-alone version of PROT-ON and its use can be reached at: https://github.com/CSB-KaracaLab/prot-on.
