# Supplementary material for: PROT-ON: A structure-based detection of designer PROTein interface MutatiONs
Source: Front Mol Biosci. 2023 Mar 1;10:1063971. doi: 10.3389/fmolb.2023.1063971 (PMC10018488; doi:10.3389/fmolb.2023.1063971)
Supplement: Supplementary file 1 [file Table1.DOCX]

Supporting Information for:

**PROT-ON: Structure-Based Detection of Critical Mutations in Redesigning Protein-Protein Interfaces**

Mehdi Kosaca^1,2^, İrem Yılmazbilek^1,3^ Ezgi Karaca^1,2*^

^1^ Izmir Biomedicine and Genome Center, Dokuz Eylul Health Campus, Izmir

^2^ Izmir International Biomedicine and Genome Institute, Dokuz Eylul University, Izmir

^3^Middle East Technical University, Ankara

*** Correspondence:**Ezgi Karaca
[ezgi.karaca@ibg.edu.tr](mailto:ezgi.karaca@ibg.edu.tr)

Keywords: interface mutation, protein-protein interaction, interface design, EvoEF1, FoldX

**SUPPLEMENTARY FIGURES**

**Figure S1.** **Box-and-whisker statistics.** Q1: The number below which 25% of the data lies; Q3: The number below which 75% of the data lies; IQR (Inter-quartile-range) = Q3-Q1 (stores 50% of the data around the median); minimum and maximum values are determined by extending the whiskers by multiple IQR (by default 1.5); negative *outliers* are the values smaller than the left whisker end, and positive *outliers* are the values greater than the right whisker end.

**Figure S2. PSSM difference scores (PSSM_diff_ = PSSM_mut_-PSSM_wt_) obtained from the iSEE training dataset (Geng et al., 2019, Proteins).** When we analyzed the experimental ΔΔG values of the iSEE data set (a refined version of SKEMPI 2.0), we noticed that the positive (most depleting, orange distribution) and negative outlier mutations (most enriching, lilac distribution) have PSSM scores =<0 and >0 respectively (Population size: 1102, positive outlier population: 25, negative outlier population: 19). Expanding on this, we aplly our PSSM filters as in the following. PROT-ON keeps a depleting designer mutation, if its PSSM score is =< 0. Complementarily, it keeps an enriching designer mutation prediction if its PSSM score is >0.


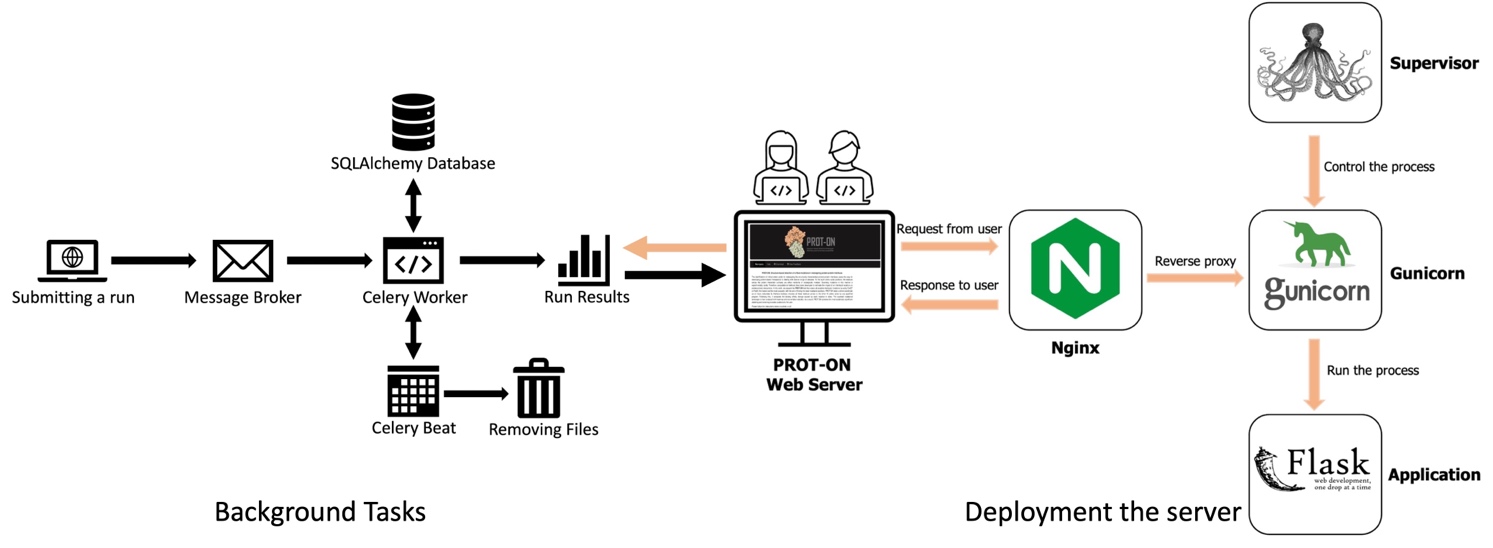


**Figure S3.** The workflow of the Celery and RabbitMQ servers to run tasks in the background and to deploy the PROT-ON web server.


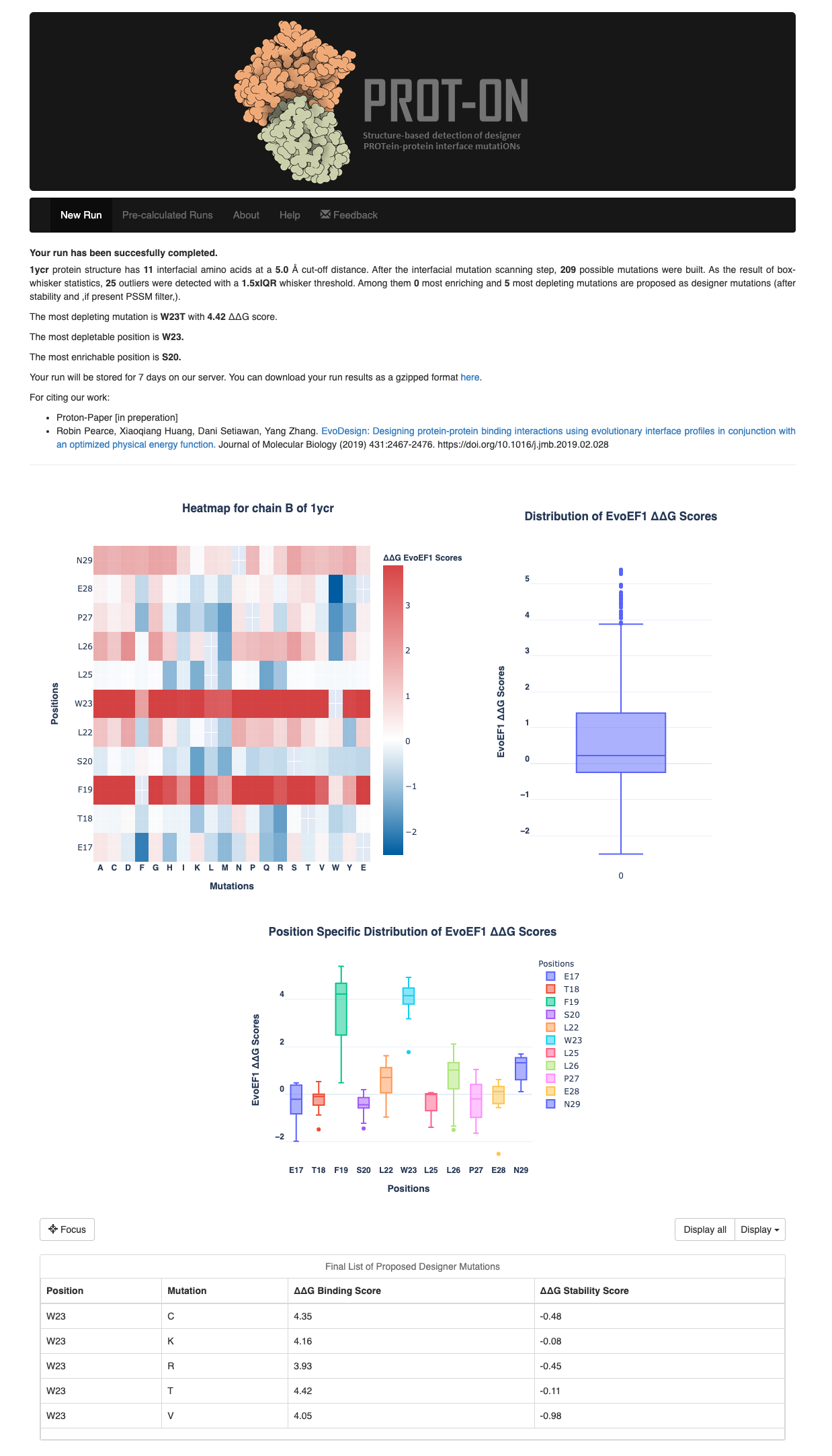


**Figure S4.** An example PROT-ON run result demonstrated on the MDM2-p53 complex. The page includes the summary on the p53 run; ∆∆G energies of interfacial mutations of EvoEF1 in the heatmap and box-and-whisker plot formats; position-specific distribution of ∆∆G energies; an interactive table of designer mutations. The run is carried out with default parameters and presented at <http://proton.tools.ibg.edu.tr:8001/result/MDM2_p53_EvoEF1>


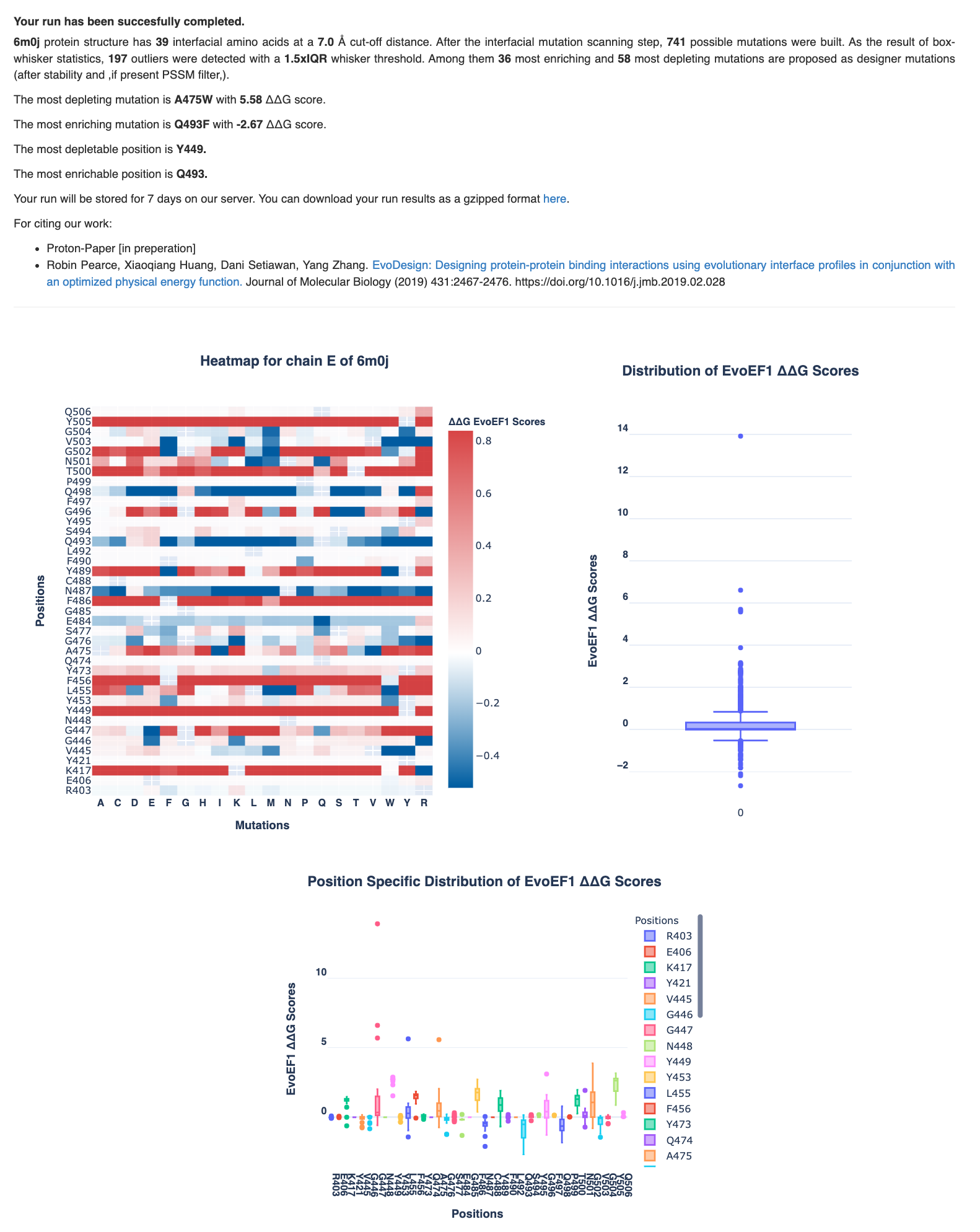


**Figure S5.** Run results for RBD monomer of ACE2-RBD complex, containing only the summary information of the run and the interactive plots. For more, please visit: <http://proton.tools.ibg.edu.tr:8001/result/ACE2_RBD_EvoEF1>


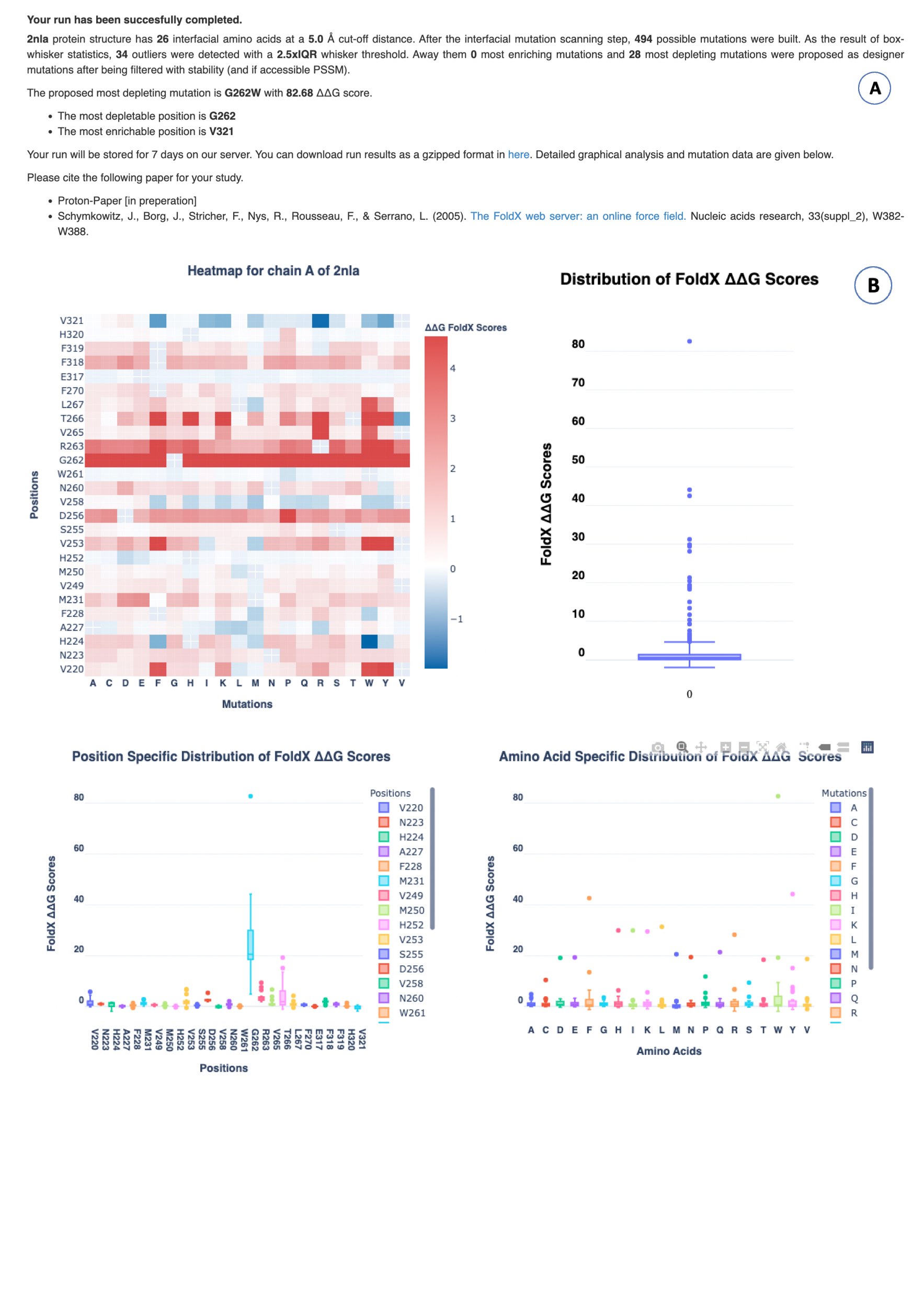


**Figure S6.** Run results for MCL1 monomer of MCL1-NOXA complex generated with the stand-alone version of PROT-ON and visualized on our web service. **(A)** Summarizing information on the MCL1 run. **(B).** Energy plots.

**SUPPLEMENTARY TABLES**

**Table S1.** Depleting designer mutations proposed by PROT-ON for p53 (with EvoEF1 and default parameters).

| Positions | Mutations | ΔΔG Binding | ΔΔG Stability |
| --- | --- | --- | --- |
| W23 | C | 4.35 | -0.48 |
| W23 | K | 4.16 | -0.08 |
| W23 | R | 3.93 | -0.45 |
| W23 | T | 4.42 | -0.11 |
| W23 | V | 4.05 | -0.98 |

**Table S2.** Enriching designer mutation list proposed by PROT-ON for RBD (with EvoEF1 and 7Å interfacial cut-off).

| **Positions** | **Mutations** | **ΔΔG Binding** | **ΔΔG Stability** |
| --- | --- | --- | --- |
| K417 | R | -0.62 | -0.26 |
| G446 | R | -0.83 | -1.13 |
| A475 | I | -0.73 | -1.61 |
| A475 | V | -0.59 | -0.93 |
| G476 | R | -1.24 | -0.4 |
| N487 | C | -0.64 | -0.63 |
| N487 | I | -0.98 | -1.36 |
| N487 | K | -1.38 | -0.65 |
| N487 | L | -0.53 | -1.83 |
| N487 | M | -2.1 | -1.12 |
| N487 | R | -0.99 | -1.33 |
| N487 | V | -0.58 | -0.56 |
| Q493 | F | -2.67 | -0.77 |
| Q493 | H | -0.7 | -0.17 |
| Q493 | I | -0.94 | -1.45 |
| Q493 | K | -1.69 | -1.14 |
| Q493 | L | -0.85 | -1.53 |
| Q493 | M | -1.82 | -0.99 |
| Q493 | R | -1.13 | -1.54 |
| Q493 | Y | -2.19 | -1.02 |
| G496 | R | -0.68 | -0.35 |
| G496 | S | -1.28 | -0.51 |
| G496 | T | -0.84 | -0.12 |
| Q498 | E | -0.52 | -0.55 |
| Q498 | F | -0.67 | -0.99 |
| Q498 | I | -1.01 | -1.41 |
| Q498 | K | -1.17 | -0.55 |
| Q498 | L | -0.74 | -1.56 |
| Q498 | M | -0.99 | -1.22 |
| Q498 | N | -0.85 | -0.05 |
| Q498 | S | -1.43 | -0.09 |
| Q498 | T | -1.82 | -0.31 |
| Q498 | Y | -0.64 | -1.19 |
| N501 | L | -0.71 | -0.61 |
| G502 | F | -0.57 | -0.41 |
| G502 | M | -0.8 | -1.72 |

**Table S3.** Depleting designer mutation list proposed by PROT-ON for MCL-1 (with FoldX, PSSM scores, and 2.5 IQR rule).

| **Positions** | **Mutations** | **ΔΔG Binding** | **ΔΔG Stability** | **PSSM Differences** |
| --- | --- | --- | --- | --- |
| V253 | F | 4.78 | -0.24 | -2 |
| V253 | W | 6.72 | -0.64 | -2 |
| V253 | Y | 6.5 | -0.07 | -3 |
| G262 | A | 4.75 | -0.25 | -4 |
| G262 | D | 19.06 | -0.49 | -5 |
| G262 | E | 19.24 | -1.46 | -1 |
| G262 | F | 42.56 | -1.57 | -6 |
| G262 | H | 29.86 | -0.26 | -2 |
| G262 | I | 29.84 | -1.34 | -4 |
| G262 | K | 29.44 | -0.96 | -5 |
| G262 | L | 31.27 | -1.31 | -5 |
| G262 | M | 20.47 | -1.04 | -5 |
| G262 | N | 19.36 | -0.27 | -3 |
| G262 | P | 11.71 | -2.69 | -3 |
| G262 | Q | 21.32 | -0.48 | -4 |
| G262 | R | 28.16 | -0.7 | -4 |
| G262 | S | 9.29 | -0.02 | -3 |
| G262 | V | 18.63 | -0.55 | -4 |
| G262 | W | 82.68 | -1.51 | -6 |
| G262 | Y | 44.16 | -1.64 | -3 |
| R263 | F | 6.38 | -1.56 | -10 |
| R263 | Y | 7.54 | -1.29 | -7 |
| T266 | F | 13.4 | -1.4 | -1 |
| T266 | H | 6.26 | -0.39 | -3 |
| T266 | K | 5.5 | -0.74 | -3 |
| T266 | R | 6.72 | -0.39 | -3 |
| T266 | W | 19.16 | -1.94 | -3 |
| T266 | Y | 15.03 | -1.43 | -1 |

References

Geng, C., Vangone, A., Folkers, G.E., Xue, L.C., Bonvin, A.M., 2019. iSEE: Interface structure, evolution, and energy‐based machine learning predictor of binding affinity changes upon mutations. Proteins: Structure, Function, and Bioinformatics 87, 110–119.
